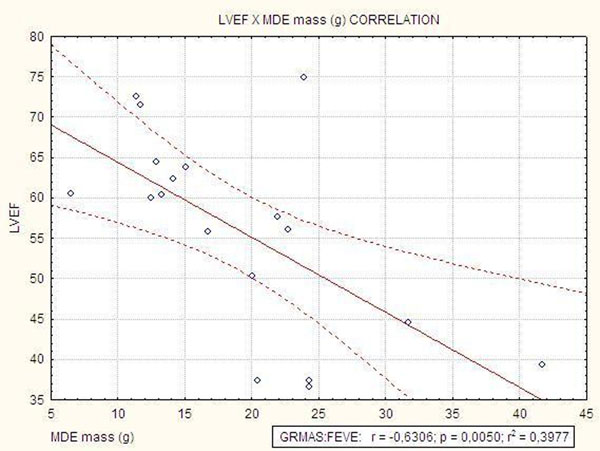# Cardiac magnetic resonance in acute rheumatic fever

**DOI:** 10.1186/1532-429X-15-S1-O23

**Published:** 2013-01-30

**Authors:** Diana Lamprea Sepulveda, Eveline B Calado, Eugênio Albuquerque, Alfredo Rodrigues, Maria Eduarda M Siqueira, Cleusa Lapa, Lurildo Saraiva, Roberta Mochiduky, Dario Sobral, Marly Uellendahl

**Affiliations:** 1 Cardiology, UNIFESP, São Paulo, Brazil; 2 Cardiology, IMIP, Recife, Brazil; 3 Radiology, Delboni/DASA, São Paulo, Brazil; 4 Cardiology, Real Hospital Portugës, Recife, Brazil; 5 Cardiology, UFPE, Recife, Brazil; 6 Cardiology, Procape-UPE, Recife, Brazil

## Background

Acute rheumatic carditis is a pancarditis in which the involvement of the pericardium and endocardium is usually confirmed by echocardiography. However, the myocardium involvement has been controversial and difficult to diagnose. We think that Cardiac Magnetic Resonance adds important information about the involvement of the myocardium in acute rheumatic fever, especially for myocardial delayed enhancement(MDE) that can detect myocardial inflammation and injury.

## Methods

We enrolled patients admitted between January and December 2010 in two different hospitals of the northeastern of Brazil with acute rheumatic fever. The patients were diagnosed according to the revised Jones Criteria for acute rheumatic fever and the World Health Organization criteria for rheumatic carditis. All the patients underwent cardiac magnetic resonance, using a1.5 Tesla scanner (Espree-Siemens, Muenchen, Germany) in two pulse sequences: a cine magnetic resonance- SSFP( Steady State Free Precession) to evaluate cardiac morphology and ventricular function, and myocardial delayed enhancement with a Phase-Sensitive Inversion Recovery (PSIR) sequence, after 0,02 mmol/Kg of gadolinium infusion to evaluate myocardial involvement. The images were analyzed by CMR-42 software.

## Results

We evaluated 18 patients with a mean age of 14,44 (±4,80) years old, 9 males and 9 females. There was no significant differences of age between males (14.88 ± 5.4 years) and females (14.00± 4.3 years), p = 0.68. Cardiac magnetic resonance imaging identified pericardial effusion and mitral valvular thickening in all 18 patients (100%), aortic valvular thickening in 17 patients (94,44%) right ventricular dysfunction in 8 patients (44,44%) and lef ventricular dysfunction in 5 patients (27,77%). MDE was present in all the patients and was characterised by a diffuse, mesocardial and heterogeneous pattern. We observed a negative correlation between MDE mass and left ventricular ejection fraction (r = −0,63; p=0,0050). In regard to gender we found a higher average of MDE mass in males( 23,90± 8,86) compared to females(14,44±4,66),p=0,0151; with no significant statistical difference on the global myocardial mass evaluated(p=0,054). Right ventricular dysfunction is associated with pulmonary hypertension evaluated by echocardiography(p=0,0415).

## Conclusions

Cardiac magnetic resonance imaging with gadolinium identified a typical inflammation pattern of MDE in acute rheumatic carditis and a negative correlation of the MDE mass and left ventricular function. Besides the small number of patients the gender differences of MDE mass may represent a more extensive inflammatory involvement in males.

## Funding

Brazilian Society of Cardiology.

**Figure 1:**
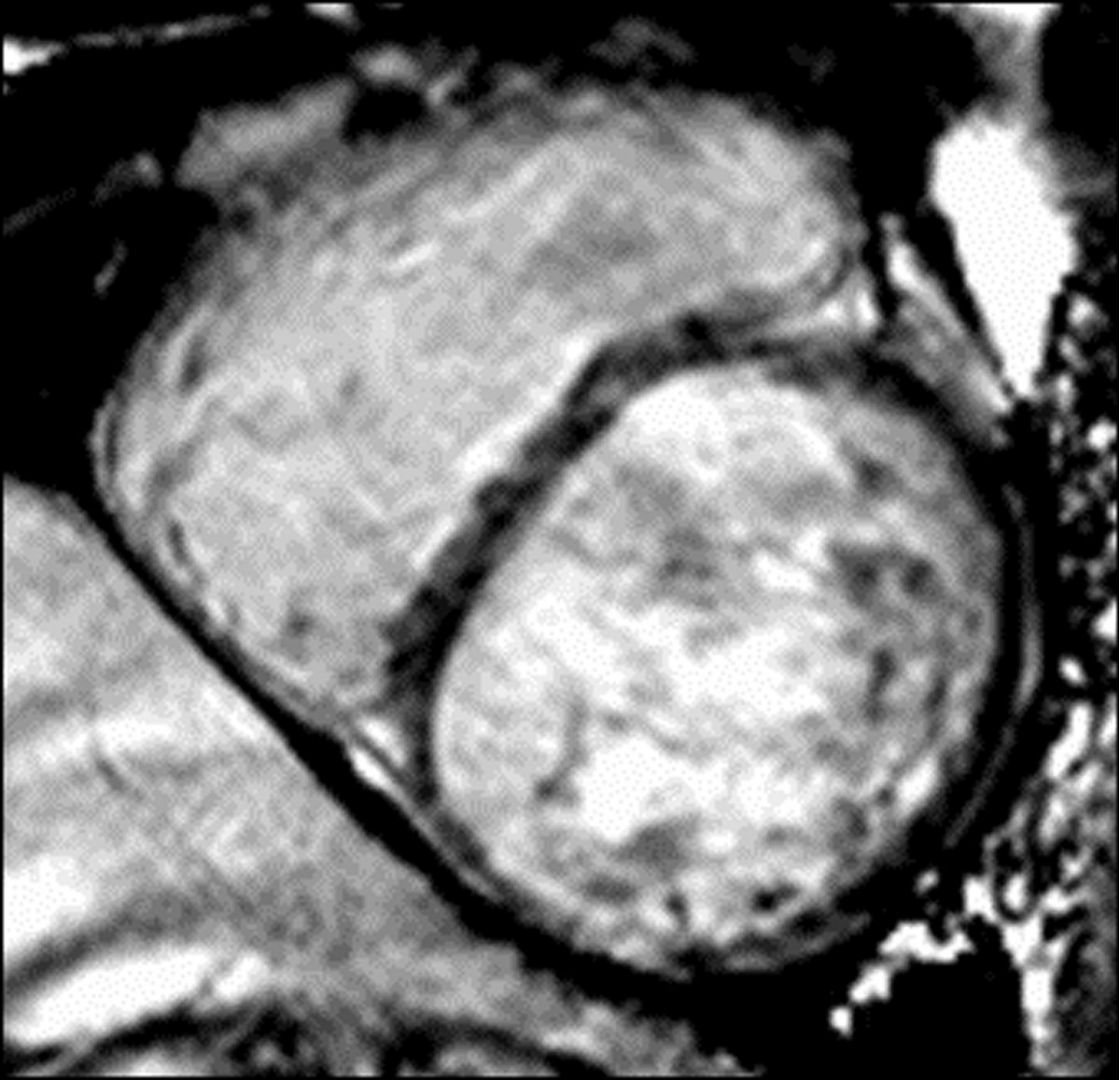


**Figure 2:**